# Uncovering the Neuroprotective Mechanisms of Curcumin on Transthyretin Amyloidosis

**DOI:** 10.3390/ijms20061287

**Published:** 2019-03-14

**Authors:** Nelson Ferreira, Maria João Saraiva, Maria Rosário Almeida

**Affiliations:** 1 Danish Research Institute of Translational Neuroscience, Nordic EMBL Partnership for Molecular Medicine, Department of Biomedicine, Aarhus University, 8000 Aarhus C, Denmark; nelson@biomed.au.dk; 2 Molecular Neurobiology Group, IBMC-Instituto de Biologia Molecular e Celular, Universidade do Porto, 4200-135 Porto, Portugal; mjsaraiv@ibmc.up.pt; 3 Instituto de Investigação e Inovação em Saúde (I3S), Universidade do Porto, 4200-135 Porto, Portugal; 4 Molecular Biology Department, ICBAS-Instituto de Ciências Biomédicas Abel Salazar, Universidade do Porto, 4050-313 Porto, Portugal

**Keywords:** curcumin, transthyretin, amyloidosis, protein aggregation, protein misfolding, drug discovery

## Abstract

Transthyretin (TTR) amyloidoses (ATTR amyloidosis) are diseases associated with transthyretin (TTR) misfolding, aggregation and extracellular deposition in tissues as amyloid. Clinical manifestations of the disease are variable and include mainly polyneuropathy and/or cardiomyopathy. The reasons why TTR forms aggregates and amyloid are related with amino acid substitutions in the protein due to mutations, or with environmental alterations associated with aging, that make the protein more unstable and prone to aggregation. According to this model, several therapeutic approaches have been proposed for the diseases that range from stabilization of TTR, using chemical chaperones, to clearance of the aggregated protein deposited in tissues in the form of oligomers or small aggregates, by the action of disruptors or by activation of the immune system. Interestingly, different studies revealed that curcumin presents anti-amyloid properties, targeting multiple steps in the ATTR amyloidogenic cascade. The effects of curcumin on ATTR amyloidosis will be reviewed and discussed in the current work in order to contribute to knowledge of the molecular mechanisms involved in TTR amyloidosis and propose more efficient drugs for therapy.

## 1. General Introduction

Transthyretin (TTR) is a plasma protein that functions mainly as a transporter for thyroid hormones, in particular thyroxine (T_4_) and retinol (vitamin A) in complex with retinol binding protein (RBP) [1]. TTR is also known to interact with other protein ligands and small molecules, either natural or synthetic compounds. In plasma, TTR interacts with apolipoprotein AI (apo A-I) [2], with the receptor of advanced glycation end-products (RAGE) [3] and with metallothionein [4]. In the cerebrospinal fluid (CSF), TTR interacts with neuropeptide Y (NPY) [5] and with amyloid-β (Aβ) peptide, indicating a neuroprotective role for TTR in the central nervous system [6,7].

Concerning small ligands, TTR binds various types of compounds [8] besides T_4_ and retinol. It binds pterins [9], halogenated polyphenols [10] and pharmacologic agents, such as some non-steroid anti-inflammatory drugs (NSAIDS) [11] and natural polyphenols of plant origin [12,13,14,15].

In humans and rodents, TTR is mainly synthetized by the liver and the choroid plexus of the brain [16,17] and is secreted to the plasma and cerebrospinal fluid, respectively [18]. In minor amounts, TTR is also synthesized in other tissues, such as the retinal pigmented epithelium, intestine, pancreas, and meninges [19,20].

At the molecular level, TTR is composed of four identical subunits of 127 amino acids forming a tetramer [21,22]. Each polypeptide chain is organized in eight segments with a β-chain structure and only a very small segment of alpha helix. The four monomers in the tetramer interact with each other through non-covalent bonds, establishing a strong interaction between two monomers, forming dimers that assemble as a tetramer originating a central hydrophobic channel limited by amino acids from both dimers. This channel has two similar binding sites for thyroxine molecules [23]. The binding sites can also accommodate other small TTR ligands that might occur in plasma as a result of metabolism, diet origin, or even compounds administered for therapeutic purposes. For an extensive review of TTR-ligand complex X-ray crystal structures, see a review by Pallaninathan et al. [24].

The predominance of the β-chain structure in the polypeptide chains of the TTR tetramer, and its organization as β-sheets contribute to the intrinsic amyloid potential of the protein, leading to aggregation, fibril formation, and deposition under specific conditions, originating transthyretin amyloidosis (ATTR amyloidosis).

## 2. ATTR Amyloidosis

ATTR amyloidosis is a systemic amyloidosis of hereditary or non-hereditary origin. The hereditary forms of the disease are due to mutations in the *TTR* gene that originate variants with a single amino acid substitution [25,26] (Available online: amyloidosismutations.com). In the non-hereditary forms, the main component of the amyloid fibrils is the wild type protein. In both cases, for different reasons, namely amino acid alterations and/or environmental conditions, TTR becomes less stable and dissociates into monomers that are partially unfolded and present a high tendency to aggregate and form fibrils that deposit in the extracellular space. More than 120 TTR variants have been described until now, related with different hereditary forms of ATTR (ATTRv). Though these are mainly systemic forms of the disease, the most affected tissues or organs where amyloid gets deposited are the peripheral nerves, gastro intestinal system, kidney, heart, carpal tunnel, eye, and in less cases the meninges [27]. The non-hereditary form of the disease is mainly associated with cardiomyopathy of aged people, over 80 years old, and the deposits are composed of wild type protein (ATTR wt) [28]. The most frequent TTR variant is TTR V30M that causes ATTRV30M amyloidosis (formerly designated familial amyloid polyneuropathy (FAP)) [29]. The disease occurs in several foci in the world, the biggest ones located in Portugal, Sweden, Japan, Brazil, Italy, France, and USA [30]. Concerning the hereditary forms of the disease, TTR V122I is also a very frequent variant, in particular, in the Black American population, being this variant related with a predominant involvement of the heart [31,32], now designated as ATTR amyloidosis with cardiomyopathy [33].

The clinical expression of the disease is highly heterogeneous in ATTR amyloidosis. In particular the age of onset of the disease is variable for different variants and even for patients with the same TTR variant, namely TTR V30M, in which the onset can vary from the 2nd to the 6th decade of life [34,35]. Early onset cases are mainly characterized by predominant loss of small-diameter nerve fibers, severe autonomic dysfunction, and cardiac conduction alterations, resulting in peripheral neuropathy leading to loss of sensation, to pain and heat, lower and upper members muscle atrophy, gastro-intestinal disturbances, and cardiomyopathy. In contrast, late onset TTR V30M patients show loss of both small and large fibers, less severe polyneuropathy, mild autonomic dysfunction and frequent cardiomegaly [36]. Among different TTR variants, there is also high variability of predominance of polyneuropathy or cardiomyopathy as main clinical manifestations in ATTRv amyloidosis (reviewed in Reference [26,37]).

## 3. Inhibitors of TTR Aggregation: Pharmacologic and Natural Inhibitors of TTR Amyloidosis

Since plasma TTR is mainly synthesized by the liver, liver transplant has been one of the first therapeutic approaches proposed and found effective for the disease [38]. However, as expected, liver transplant is an invasive therapy, not suitable for all patients and with several limitations and risks [39]. In addition, recently, it was found that after liver transplant, some patients develop TTR cardiomyopathy due to deposition of wild-type TTR in their heart [40,41,42]. This supports the need for alternative therapeutic approaches that aim to stabilize TTR using small molecules that, by binding to TTR, stabilize it and inhibit its aggregation and deposition [43]. The first evidence of TTR stabilization through binding of small compounds came from the fact that when TTR is bound to T_4_ it is less prone to aggregation. In addition, T_4_ binding sites in TTR are mostly unoccupied due to the high TTR/T_4_ ratio in plasma, allowing TTR stabilization by binding of small compounds to TTR with high affinity [44].

Several nonsteroidal anti-inflammatory drugs (NSAIDs), have been known for a long time to compete with T_4_ for the binding to TTR, such as salicylates, diclofenac, flufenamic acid and diflunisal [45]. Among these, diflunisal was one of the most promising compounds due to its affinity and specificity to bind TTR. In addition, several diflunisal derivatives have been synthetized to improve its affinity and selectivity to bind TTR in plasma [46,47]. Diflunisal is still one of the compounds in use for ATTR amyloidosis therapy in countries where Tafamidis has not yet been approved [48,49]. Tafamidis, diclorofenol benzoxazole carboxylic acid, is a more recent and widely-used drug that binds to TTR and stabilizes it [50,51]. Tafamidis is highly safe and tolerable and has been found efficient in slowing disease progression and preserving quality of life of TTR V30M patients [52]. Meanwhile, other strategies for ATTR amyloidosis therapy have also been pursued, namely targeting different steps in the cascade of amyloid formation, fibril disruption, and clearance [53]. An example of such strategies is to use compounds that bind to TTR and block its polymerization or disrupt the amyloid fibrils formed, such as molecular tweezers (CLR01) and doxycycline, respectively [54,55].

## 4. Natural Inhibitors—Polyphenols

In the search for compounds of therapeutic interest, presenting very low toxicity and structural similarities to other TTR ligands, several polyphenols of plant origin have been studied as inhibitors of TTR amyloidogenesis. Some polyphenols were previously reported as inhibiting protein aggregation and amyloid formation in neurodegenerative diseases, such as Alzheimer’s and Parkinson’s disease [56]. One of the most studied polyphenols is resveratrol. In vitro studies using the AC16 cardiomyocyte cell line demonstrate that resveratrol is able to stabilize the native TTR tetramer, preventing the formation of cytotoxic species and promoting aggregation of monomeric into non-toxic species [12]. Furthermore, administration of resveratrol to Alzheimer’s disease (AD) mice revealed an increase in TTR levels in plasma that does not result from higher expression of the protein, but, instead, might be related with increased TTR stability and longer half-life in circulation [57]. However, resveratrol seems to have not only these direct effects on TTR but also other properties namely those involving protection against oxidation, which is difficult to discern.

Other polyphenols, like nordihydroguaiaretic acid (NDGA), rosmarinic acid, caffeic acid and epigallocatechin gallate (EGCG), have also been investigated in vitro for their interaction with TTR [58,59,60,61,62]. Contrary to most polyphenols, EGCG did not compete with T_4_ for binding to TTR, revealing that it binds at a different binding site in the molecule [62]. Indeed, the crystallographic structure of the complex of TTR with EGCG revealed that it binds at different regions at the surface of the molecule and not at the T_4_ binding sites [63]. In a subsequent structure–activity study, the galloyl moiety has been highlighted as a key structural feature of EGCG by greatly enhancing its anti-amyloid chaperone activity of TTR [61].

When administered to a model mice expressing human TTR V30M, EGCG inhibited TTR deposition in the gastrointestinal tract and in the dorsal root ganglia (DRG), the main sites of aggregated TTR deposition in this animal model for the disease [64]. In addition, when administered to old mice, EGCG treatment resulted in a decrease of TTR deposits in tissues, indicating a disruptive effect on aggregated TTR deposits. A small pilot study with EGCG administration to human carriers of amyloidogenic TTR mutations including TTR V30M revealed a reduction of myocardial mass in the case of cardiomyopathy, indicating an inhibitory effect of EGCG on TTR amyloid fibril formation [65,66]. The reported studies show improvement in the cardiac function without increase of the patient’s survival. The low toxicity and high tolerability to EGCG, confirmed in these studies, encourage continuation of treatment with EGCG [67].

Among the polyphenols studied in vitro, curcumin revealed a particular behavior suggesting different mechanism of inhibition of ATTR amyloidosis [58].

## 5. In Vitro Studies with Curcumin

### 5.1. Curcumin Binds to TTR and Increases Its Resistance to Dissociation

A decade ago, Pullakhandam and colleagues first reported curcumin interaction with TTR [68]. Using Scatchard analysis of fluorescence quenching, the authors showed that curcumin binds to wild-type TTR with a molar ratio of 1.2:1 and *K*_d_ of 2.3 × 10^−6^ M [68]. In addition, curcumin was found to dose-dependently displace 1-anilino-8-naphalene sulfonate (ANS) at pH 7.2 from TTR’s central ligand-binding channel, to which various ligands are known to bind [68].

Shortly after, we further detailed the interaction between curcumin and TTR by unequivocally showing that curcumin competed with radiolabeled T_4_ ([^125^I]T_4_) for its binding to wild-type and V30M mutant TTR, both in vitro and in whole human plasma [58]. These observations were later corroborated by the crystal structures of TTR complexes with curcumin and also its degradation product, ferulic acid, [15], and other curcumin-like compounds [69], showing that curcumin interacts with Ser 117 and Lys15 and with Val 121 and Thr123 through a water molecule [15]. By filling the largely unoccupied T_4_ binding pockets at the weaker dimer–dimer interface, curcumin increases TTR tetramer resistance to dissociation in non-native monomers as shown by isoelectric focusing (IEF) studies in semi-denaturing conditions (4 M urea) [58]. This, together with selective binding of curcumin to TTR over other plasma proteins, resulted in a 25% increase of the tetramer/total TTR ratio in plasma from controls and TTR V30M heterozygote carriers [58].

### 5.2. Curcumin Redirects TTR Aggregation into “Off-Pathway” Oligomers and Disaggregates Pre-Formed TTR Amyloid Fibrils

Despite its inability to prevent the acid induced aggregation of TTR wild-type [68], we have shown that curcumin robustly inhibits aggregation of the highly amyloidogenic Y78F variant under physiological conditions (phosphate buffered saline, pH 7.4, 37 °C) [58]. This supports the hypothesis that protonation and isomerization of the phenolic and enolic hydroxyl groups of curcumin at low pH might impair interaction with TTR [68]. Under transmission electron microscopy (TEM) and dynamic light scattering (DLS), curcumin redirected the TTR Y78F amyloid formation pathway into a monodispersed, highly stable population of “off-pathway” oligomers with approximately 80 nm in hydrodynamic diameter (dH) [58]. In addition, we found that Schwann cells exposed to TTR Y78F aggregates incubated with curcumin presented significantly reduced endoplasmic reticulum (ER) stress and were protected from entering into the apoptotic signaling pathway [70], highlighting that curcumin-induced oligomers are less toxic than untreated “on-pathway” aggregate intermediaries. Moreover, dot–blot analysis of conditioned medium from Rat Schwannoma (RN22) cells expressing TTR L55P incubated with curcumin revealed almost complete inhibition of TTR aggregation (90%). This variant is associated with an aggressive form of ATTR amyloidosis, further supporting the protective role of curcumin on the early stages of TTR aggregation, either by inhibiting tetramer dissociation and/or redirecting pathological misfolding and aggregation into more innocuous counterparts [58]. Similar observations were later reported relative to different aggregation-prone proteins associated with neurodegeneration, including Aβ [71], tau [72] and α-synuclein [73].

Beyond sharing many structural similarities with classical amyloid-binding dyes, such as Thioflavin-S, Congo red, and crysamine-G, curcumin showed specific labeling of amyloid deposits [74,75,76]. Although the precise atomic-detailed characteristics underlying the ability of curcumin to break down β-sheet rich aggregates remain unclear, solid-state NMR studies have highlighted the structural importance of the aromatic carbons adjacent to the methoxy and/or hydroxy groups of curcumin in its binding with Aβ fibrils [77].

Overall, multiple lines of evidence favor the hypothesis that the non-specific modulatory role of curcumin on amyloid formation and toxicity in vitro depends on aggregate-related conformational structure rather than protein primary sequence.

## 6. In Vivo Studies with Curcumin

### 6.1. Curcumin Reduces TTR Load and Degrades Amyloid Deposits in Tissues

In recent years, an increasing amount of evidence supporting the anti-amyloidogenic role of curcumin in different proteins prone to misfolding have paved the way to preclinical trials in transgenic animal models [78].

With regard to ATTR amyloidosis, we have shown that chronically feeding young transgenic mice for human TTR V30M with curcumin (2% *w*/*w*) results in micromolar steady-state levels of curcumin in plasma (21.4 ± 3.6 μM) [79]. Selective competition of curcumin with T_4_ (42%) for the binding to TTR in plasma significantly reduced tetramer dissociation into non-native monomeric intermediaries under semi-dissociating conditions [79]. Beyond stabilizing TTR native fold, curcumin supplementation alleviated TTR load and associated biomarkers in the gastrointestinal tract, the primary target organ in this mouse model [79]. Dietary intake of curcumin was well-tolerated and non-toxic to animals and the treatment did not interfere with TTR plasma levels in vivo [79].

In a later study, we evaluated the effect of curcumin in aged mice expressing the TTR V30M variant on an *Hsf-1* heterozygous background (hTTR V30M/Hsf), in which deposition of aggregated TTR coexists with birefringent congophilic material in tissues. We found that curcumin intake not only reduced non-fibrillar extracellular TTR burden in both gastrointestinal tract and dorsal root ganglia, but also remodeled pre-existing congophilic amyloid material in tissues [70].

Our findings are in close alignment with recent observations made by others showing that curcumin promotes remodeling of existing amyloid deposits and counteracts the formation of new amyloid deposits, or even reduce the amount of remaining deposits [72,76,78,80].

### 6.2. Other Neuroprotective Mechanisms of Curcumin

**Figure 1:**
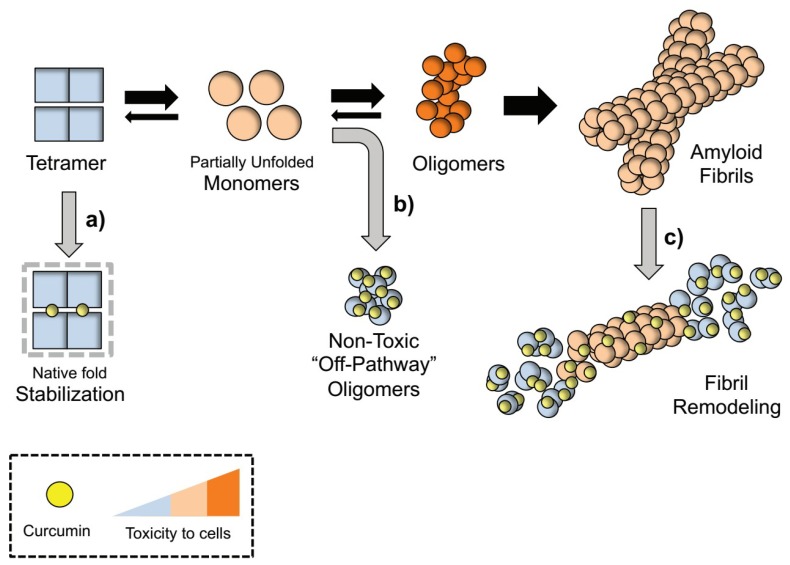
Proposed mechanism for TTR aggregation pathway modulation by curcumin. Rate-limiting tetramer dissociation of TTR into partially unfolded monomers precedes the formation of toxic oligomeric intermediates that evolve into β-sheets enriched mature fibrils. Curcumin modulates TTR cascade by directly interacting with different binding partners: (**a**) Curcumin interaction with TTR at the T_4_ binding pockets stabilizes the tetrameric fold and blocks its dissociation into unfolded monomeric species [15,58,68]; (**b**) Curcumin interaction with partially misfolded non-native monomers redirects TTR aggregation into “off-pathway” unstructured oligomers innocuous to cells [58,68]; (**c**) Curcumin breaks down and remodels β-sheet rich TTR fibrils in smaller amorphous aggregates in in vitro [58] and in vivo [70].

Although we hypothesize that curcumin alleviates TTR extracellular burden most likely due to its ability to directly interact and modify multiple partners of the TTR amyloid cascade, as summarized in Figure 1, we speculate whether the pleiotropic therapeutic actions of curcumin [78] might synergistically potentiate its efficacy in vivo.

Recently, increasing relevance has been attributed to endothelial abnormalities associated with ATTRv amyloidosis and in particular ATTR V30M [81,82]. It has been suggested that TTR variants may affect endothelial cells function even before amyloid fibril formation. Thus, microangiopathy could play an important role in an initial lesion leading to organ damage [83]. Interestingly, curcumin appears to improve endothelial cell function and, though its mechanisms of action are not completely known, it seems that by lowering the expression of pro-inflammatory molecules, and by reducing levels of reactive oxygen species, such as Nox-2 in endothelial cells, curcumin not only decreases trans-endothelial monocyte migration, but also maintains adequate NO levels for the proper function of cells [84].

Accumulating evidence has linked autophagy impairment to neurodegeneration and neuronal cell death [85,86]. Given that stimulation of autophagy can potentially enhance degradation of aggregation prone-proteins, development of autophagy-inducing therapies, in which toxic misfolded proteins are used as autophagy substrates, might be a valuable pharmacological approach for neurodegenerative diseases, including ATTR amyloidosis [85,86].

In preclinical studies performed with TTR V30M transgenic mice, curcumin has been shown to effectively reverse accumulation of p62, a key cargo receptor involved in selective autophagy, re-establishing the autophagic flux and mitigating apoptosis [87]. Nevertheless, since curcumin can mediate crosstalk between different signaling pathways [88,89] it remains unclear to which extent restoration of the autophagic flux in vivo occurs because: (i) curcumin promotes autophagy or (ii) its anti-amyloid activity prevents TTR “on-pathway” aggregation reaching a critical threshold beyond which the autophagic machinery would be overwhelmed and irreversibly damaged.

In recent years, macrophage-mediated clearance of amyloid by a variety of phagocytic and digestive mechanisms has been receiving increasing attention in the literature [90]. Several small-molecules, including derivatives of curcumin, have been found to promote phagocytosis of Aβ by macrophages [91,92,93]. Similarly, we have shown that pre-treatment of macrophages isolated from aged FAP mice with physiologically achievable doses of curcumin, improves phagocytic uptake and degradation of extracellular TTR aggregates, supporting that curcumin restores the inefficient macrophage TTR clearance characteristic of pathological conditions [70].

## 7. Final Remarks

Several lines of evidence suggest that curcumin has neuroprotective properties in many protein-misfolding disorders, including Alzheimer’s and Parkinson’s diseases and ATTR amyloidosis [78]. Curcumin is a biologically well-tolerated polyphenol, with a long established safety history [94]. According to JECFA (The Joint United Nations and World Health Organization Expert Committee on Food Additives) and EFSA (European Food Safety Authority) guidelines, the recommended allowable daily intake (ADI) amount of curcumin is 0–3 mg/kg body weight [94]. Nonetheless, some minor undesired side effects have been reported in a single dose escalation study where healthy subjects were given increasing doses from 0.5 to 12 g of curcumin [95].

Despite its well-documented therapeutic efficacy, the poor absorption and rapid metabolism of curcumin, has hindered its progress as a prospective pharmacological agent. To increase its bioavailability, a wide array of novel formulations have been developed, including nanoparticles, liposomes, micelles, and phospholipid complexes, which increase the bioavailability of curcumin by providing longer circulation, enhanced permeability, and resistance to metabolic degradation and excretion [78].

**Figure 2:**
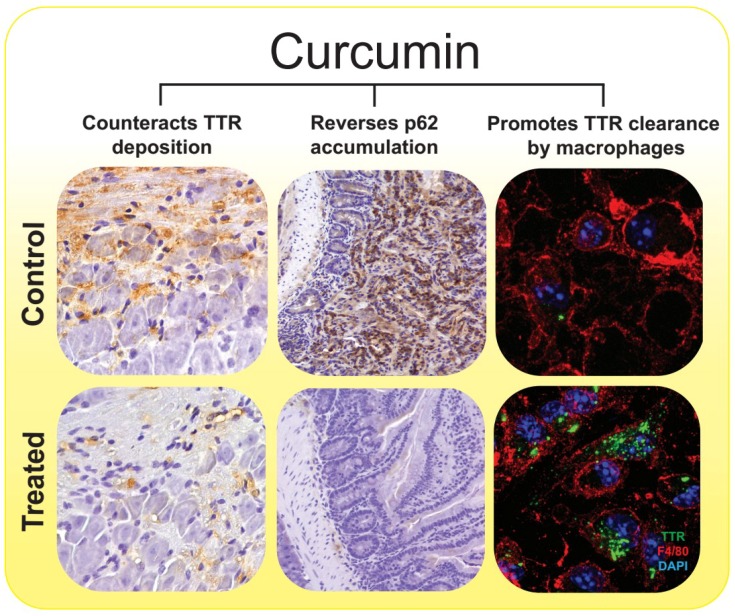
The pleiotropic effects of curcumin on the molecular pathways associated with ATTR amyloidosis. Curcumin exerts neuroprotective effects on ATTR amyloidosis by modulating TTR abnormal aggregation and counteracting TTR tissue deposition (left panels, 20× magnification) immunohistochemistry (IHC) analysis of TTR in dorsal root ganglia (DRG) from mice expressing human TTR V30M (hTTRV30M mice) treated with curcumin and age-matched controls [70]), re-establishing the autophagic flux by reversing p62 accumulation (center panels, 20× magnification), IHC analysis of p62, in duodenum samples from hTTRV30M mice treated with curcumin and age-matched controls [87]) and improving the phagocytic uptake and degradation of extracellular TTR aggregates by macrophages (right panels, 63× magnification), double immunofluorescence labeling for TTR, in green, and F4/80, in red, of primary macrophages from hTTRV30M mice that were pre-incubated in presence of curcumin or its absence (control), before addition of TTR aggregates to cell culture medium [70]). Nevertheless, other well-known neuroprotective properties of curcumin, such as its anti-inflammatory, anti-apoptotic, and anti-oxidative activities [78,94], might potentiate its in vivo effects.

Presently, numerous disease-modifying targeted therapies for TTR amyloidosis are being tested in human clinical trials, including TTR stabilizers (diflunisal, tafamidis), fibril disruptors (doxycycline/TUDCA) and the most recent gene therapies to block TTR expression (small interference RNAs (siRNAs) and antisense oligonucleotides therapy (ASOs)) [26,37,96,97]. Although development of these strategies greatly improved the perspectives in ATTR amyloidosis, the complex nature of the disease, in which several pathways are known to contribute to the pathology, prompts to seek multi-stage interventions that not only block TTR synthesis and/or misfolding, but also suppress inflammation and oxidative damage and enhance cellular protein degradation systems. Taken together, the pleiotropic activities of curcumin provide multiple ways to tackle TTR pathophysiology, either through direct interaction of curcumin with TTR, or indirect effects affecting signaling pathways associated with TTR amyloid fibril formation and clearance. Accordingly, the works here reviewed, and summarized in Figure 2, demonstrate interaction of curcumin with TTR through binding at the thyroxine binding sites, resulting in TTR tetramer stabilization and consequent modulation of the TTR misfolding cascade inhibiting aggregation and /or inducing formation of non-toxic aggregates. This leads to restoring the autophagy flux and improving phagocytic uptake and clearance of extracellular TTR. Curcumin also appears to directly induce disaggregation of TTR pre-formed fibrils and to promote clearance of TTR aggregates through endocytose by fibroblasts and macrophages. Concomitant with these effects, curcumin presents several non-specific effects counteracting common pathogenic events in amyloidosis, such as oxidative stress, inflammation, apoptosis and extracellular matrix dysregulation within a range of dosing with proven safety.

In conclusion, in this context, curcumin remains a promising scaffold for the development of potent multi-stage disease-modifying drugs for the treatment of TTR amyloidosis.
